# Kinematic alignment doesn't tell the whole story: It's time for kinetic alignment

**DOI:** 10.1002/ksa.70010

**Published:** 2025-09-09

**Authors:** Umile Giuseppe Longo, Giovanni Spallone, Arianna Carnevale, Letizia Mancini, Emiliano Schena, Rocco Papalia, Michael Tobias Hirschmann

**Affiliations:** ^1^ Fondazione Policlinico Universitario Campus Bio‐Medico Rome Italy; ^2^ Department of Medicine and Surgery, Research Unit of Orthopaedic and Trauma Surgery Università Campus Bio‐Medico di Roma Rome Italy; ^3^ Research Unit of Measurements and Biomedical Instrumentation Fondazione Policlinico Universitario Campus Bio‐Medico Roma, Università Campus Bio‐Medico di Roma Rome Italy; ^4^ University Clinic for Orthopedic Surgery and Traumatology, Kantonsspital Baselland Bruderholz Switzerland; ^5^ Department of Clinical Research, Research Group Michael T. Hirschmann, Regenerative Medicine & Biomechanics University of Basel Basel Switzerland

**Keywords:** dynamic hip‐knee‐ankle angle, kinetic alignment, kinetics, total knee arthroplasty

## Abstract

Kinematic alignment is increasingly adopted in total knee arthroplasty (TKA) as a patient‐specific strategy to restore native joint anatomy. However, its reliance on static radiographic measurements may not adequately reflect real‐world functional biomechanics. This editorial underscores the importance of complementing static assessment with kinetic principles. This emerging concept, referred to as kinetic alignment, integrates dynamic parameters such as the dynamic hip‐knee‐ankle angle, knee adduction moment, ground reaction forces, and muscle forces to better characterise in vivo joint loading. These kinetic variables provide critical insight into joint loading during real‐life activities and may offer greater predictive value for implant performance and patient satisfaction. Continued emphasis on static alignment targets may overlook key elements of in vivo knee function. Incorporating kinetic data into preoperative planning could support more tailored surgical decisions, helping to mitigate risks related to malalignment, overloading, and suboptimal outcomes. The editorial advocates for expanding the concept of alignment beyond static geometry, including both motion and load, and encouraging the orthopaedic and biomechanical communities to adopt a more functional and individualised perspective in TKA planning.

**Abbreviations**
dHKAA: dynamic hip‐knee‐ankle angle
GRF: ground reaction forces
KA: kinematic alignment
KAM: knee adduction moment
KOA: knee osteoarthritis
TKA: total knee arthroplasty

Among the proposed alignment strategies in total knee arthroplasty (TKA), kinematic alignment (KA) has gained increasing attention as a patient‐specific approach aimed at restoring the native knee anatomy and kinematics [19, 26, 28, 31, 35, 37]. However, KA is not universally suitable for all individuals [16, 20, 30, 35, 38]. Particularly, native limb alignments such as excessive varus [30, 35] may be biomechanically incompatible with prosthetic design, leading to altered load distribution, increased polyethylene wear and potential implant failure [35]. Moreover, like all currently adopted alignment strategies, KA relies on measurements derived from double‐leg, weight‐bearing, long‐leg radiographs, a static modality that captures only a single, fixed postural condition. Recent advances in robotic‐assisted surgery have enabled the implementation of functional alignment [5, 8, 14, 35], allowing dynamic intraoperative adjustment of implant positioning based on soft tissue balance. This technique represents a significant step forward compared to traditional bone‐referenced approaches. However, all current alignment approaches do not account for knee dynamic behaviour [12, 16, 18, 24] during functional tasks, limiting their ability to accurately reflect real‐world joint loading conditions. Dynamic activities such as walking, stair climbing, or sit‐to‐stand transitions involve continuous changes in limb alignment and joint loading [9, 11, 36, 39], underscoring the need for evaluation methods that capture not only joint motion but also the associated loading patterns. Several longitudinal studies emphasise this weakness, failing to demonstrate a significant association between static alignment and TKA longevity [1, 6, 9, 33].

To overcome the inherent limitations of static imaging, recent literature has increasingly focused on dynamic alignment. This emerging approach aims to describe how lower limb alignment evolves throughout the gait cycle, rather than relying on a single static posture. In this scenario, the dynamic hip‐knee‐ankle angle (dHKAA), obtained through three‐dimensional motion analysis, has been suggested as a valuable metric [9, 13, 21, 36, 39]. Notably, these studies have demonstrated that static alignment is not predictive of dynamic alignment patterns during gait cycle [9, 13, 36, 39], nor of the post‐operative knee adduction moment (KAM) [13, 32, 36], which is widely recognised as a key index of medial knee compartment loading and KOA progression [2, 3, 4, 22, 40]. These findings emphasise the limitation of using only static parameters to understand knee biomechanics [17, 18], particularly in TKA planning. Conversely, dynamic alignment could provide additional insights into medial and lateral compartments knee load [13, 25], potentially acting as a predictor of prosthetic longevity.

However, kinematics tells us how we move, but kinetics explains why, since kinematics describes motion through joint angles and movement over time, while kinetics deals with the forces and moments that give rise to motion and determine joint loading.

To characterise the in vivo loading conditions on the knee during daily activities, joint moments, ground reaction forces (GRFs), and muscle forces are critical. Joint moments are crucial as they are indexes of the mechanical demand on the knee joint resulting from GRFs (external moments) or muscle activity (internal moments) [7]. Among them, special attention should be given to the KAM, as previously mentioned, since it represents the tendency of the knee to move into varus or valgus alignment, directly mirroring medial knee compartment loading [2, 3, 4, 22, 40]. On the other hand, the magnitude, direction and point of application (i.e., centre of pressure) of the GRF may be useful in the determination of compensatory movement strategies and risk of overloading specific knee joint compartments [10, 27]. Additionally, muscular contractions, particularly from the quadriceps, hamstrings, and gastrocnemius, play an important role in modulating joint loading, contributing to both joint compression and dynamic stability [15]. Following TKA, alterations in muscle recruitment patterns, such as persistent quadriceps weakness and compensatory hamstring overactivation [34], may lead to asymmetric or excessive loading, potentially compromising implant longevity and functional recovery. For instance, imbalances within the quadriceps, including weakness of the vastus medialis and dominance of the vastus lateralis, have been linked to lateral patellar maltracking [23, 34]. Moreover, weakness of the hip abductors and external rotators, frequently seen in patients with concomitant hip osteoarthritis, may contribute to dynamic valgus alignment and increase lateral compartment loading [29, 34]. These altered loading patterns can exacerbate polyethylene wear, promote instability, and ultimately affect implant survival. Evaluating only joint angles, while ignoring kinetic parameters (i.e., joint moments, GRFs and muscle force) may lead to incomplete or even clinically inadequate interpretations. The integration of kinetic data is fundamental in the development of realignment strategies that are not only personalised but also tailored to each patient's functional demands. An evolution of alignment assessment methods from static radiographic imaging to dynamic and kinetic evaluations is shown in Figure 1.

**Figure 1:**
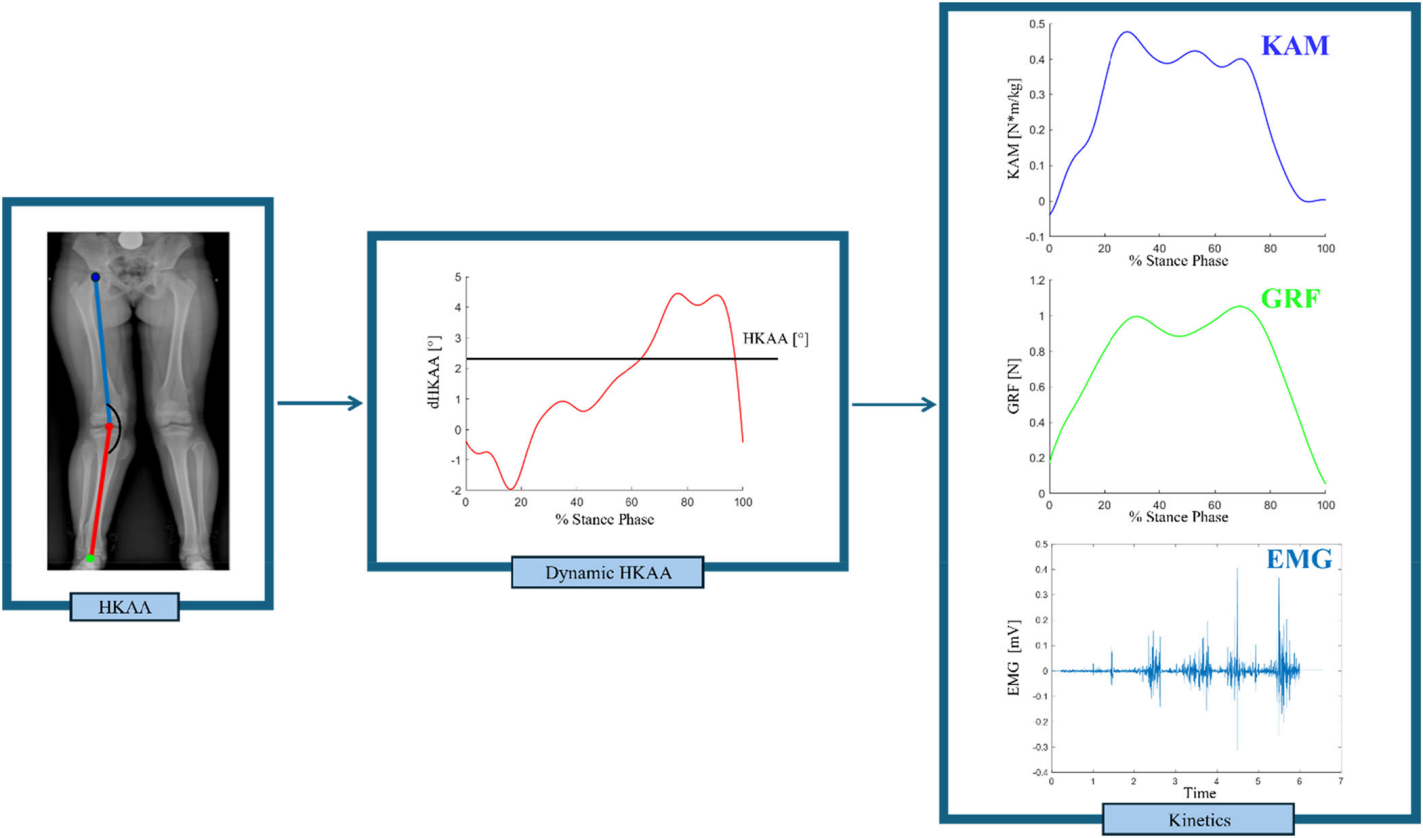
Overview of alignment assessment progressing from static to dynamic and kinetic analysis. Left: Static HKAA from long‐leg radiograph. Centre: Dynamic HKAA across the stance phase, captured via three‐dimensional motion analysis. Right: Kinetic parameters derived from gait analysis, KAM (top), GRF (middle), and EMG (bottom), offering insight into real‐world joint loading and neuromuscular control during movement. EMG, electromyography; GRF, ground reaction forces; HKAA, hip‐knee‐ankle angle; KAM, knee adduction moment.

In conclusion, improving long‐term outcomes after TKA requires moving beyond the static concept of ‘correct alignment option’. In the current and ongoing debates about the best alignment technique, it is finally time to integrate static images with dynamic and kinetic insights. Future applications may involve the use of kinetic metrics, obtained for instance from gait laboratories, to support advanced preoperative planning tools and intraoperative navigation platforms.

These gait labs, traditionally limited to observational analysis, could evolve into decision‐support environments where dynamic loading data helps inform surgical planning and implant optimisation. This approach may allow clinicians to better predict prosthesis behaviour, identify high‐risk patients earlier, and improve surgical planning. The orthopaedic and biomechanical community are encouraged to consider this emerging perspective since movement is not only a matter of angles but also of forces. Only with time and research will it become clear whether a more personalised, kinetic‐based approach to TKA alignment leads to improved clinical outcomes and greater patient satisfaction.

## AUTHOR CONTRIBUTIONS

All authors were equally involved in the conceptualisation, draughting, and final approval of the editorial.

## CONFLICT OF INTEREST STATEMENT

The authors declare no conflicts of interest.

## ETHICS STATEMENT

None declared.
